# Do children’s feeding practices affect growth outcomes in sub-Saharan Africa? A multi-country study

**DOI:** 10.1186/s41043-026-01250-9

**Published:** 2026-02-21

**Authors:** Bedilu Alamirie Ejigu, Khadijat Adeleye, Linus Baatiema, Edward Kwabena Ameyaw, Sanni Yaya

**Affiliations:** 1 Department of Statistics, CNCS, Addis Ababa University, Addis Ababa, Ethiopia; 2 Elaine Marieb College of Nursing, University of Massachusetts Amherst, Amherst, USA; 3 Ghana Health Service, Upper West Region, Wa, Ghana; 4 Centre for Migration, Security and International Relations, University of Business and Integrated Development Studies, Box 64 Wa, Ghana; 5 45L & E Research Consult Ltd, Upper West Region, Wa, Ghana; 6 School of Graduate Studies and Institute of Policy Studies, Lingnan University, Tuen Mun, Hong Kong; 7 Faculty of Medicine, University of Parakou, Parakou, Benin; 8 The George Institute for Global Health, Imperial College London, London, UK

**Keywords:** Child malnutrition, Growth outcomes, Feeding practices, Sub-Saharan africa, Low birth weight

## Abstract

**Introduction:**

Sub-Saharan Africa faces unique nutritional challenges, exacerbated by conditions such as poverty, limited access to healthcare, and diverse dietary traditions. Despite the scholarship on children’s feeding and their nutritional conditions in sub-Saharan Africa, scanty evidence exists on the association between children’s feeding practices and growth outcomes. Consequently, this study seeks to provide a nuanced understanding of children’s feeding practices and their association with growth outcomes in sub-Saharan Africa.

**Methods:**

Demographic and Health Survey data from 30 countries were used for this study, with a sample aged 0–59 (*n* = 174,281). The descriptive analysis involved the computation of child and maternal factors, presented in frequencies and percentages. We further analysed the weighted percentages of stunting, wasting, and underweight and their co-existence across included countries and children’s socio-demographic characteristics. To investigate the association between child feeding practices and other demographic factors with child weight and growth outcome, we employed a multilevel logistic regression.

**Results:**

We noted that 6.94% (95%CI:6.79,7.09) were wasted, 32.17(95%CI:31.90,32.45) were stunted, 16.72%(95%CI:16.50,16.94) were underweight children, and 2.32% (95%CI:2.24,2.40) were wasted, stunted and underweight. Age-appropriate feeding (conceived as not wasted, stunted or underweight based on WHO’s definition) increased co-occurrence risk (AOR = 1.68), while exclusive breastfeeding reduced it (AOR = 0.40). Children with low birth weight were more likely to be wasted [AOR = 1.65, 95% CI = 1.57–1.74], stunted [AOR = 1.49, 95% CI = 1.44–1.54] and underweight [AOR = 1.91, 95% CI = 1.84–1.98]. Females were observed to have lower odds of all three indicators as manifested in their co-existence (AOR = 0.59, 95% CI = 0.55–0.63). Children 12–23 months had the highest odds of experiencing the co-existence of stunting, wasting and underweight (AOR = 5.86, 95% CI = 4.94–6.95) relative to those below 6 months.

**Conclusion:**

Feeding practices significantly predict growth outcomes, urging policies for age-appropriate diets, dietary diversity, and breastfeeding support. The findings underscore the urgent need to address child malnutrition, particularly stunting, wasting, and underweight, to improve child health and well-being. These findings should inform policymakers, healthcare professionals, and stakeholders in developing effective strategies to improve child nutrition and overall well-being in the region. Context-specific policies and interventions, prioritizing maternal nutrition, access to quality prenatal care, and appropriate infant feeding practices, are crucial in mitigating the impact of malnutrition on children in the region. Further research and collaboration are needed to develop targeted strategies and sustainable solutions to tackle child malnutrition effectively in sub-Saharan Africa.

## Introduction

Malnutrition is a pressing global health issue, with its most severe impact felt in economically disadvantaged regions. According to the 2017 report of the Food and Agriculture Organization (FAO), approximately 821 million people worldwide suffer from various forms of malnutrition [1]. Asia and Africa carry the heaviest burden, with children being disproportionately affected [2]. SSA has the highest global burden of child malnutrition: 34% stunting, 6% wasting [3]. Stunting, wasting, and underweight are well-established indicators of infant and child growth, reflecting their overall health and well-being [4–6]. Stunting, expressed as height-for-age, is a robust marker of impaired growth and the most prevalent form of child malnutrition. It is a slow, cumulative process that typically becomes permanent if established [6]. Wasting, defined as low weight-for-height, often indicates severe and recent weight loss, frequently associated with inadequate nutrition and prolonged illnesses (6). Underweight signifies low weight-for-age [7] .

Beyond their clinical and anthropometric manifestations, these forms of undernutrition also come with substantial economic consequences at individual, household, and societal levels [8] Children who are stunted, wasted or underweight are more likely to experience delayed cognitive development, poorer school readiness and attainment, and reduced productivity and earnings in adulthood, thereby perpetuating intergenerational cycles of poverty and vulnerability [9, 10].These long-term losses are linked to everyday infant and young child feeding behaviours in sub-Saharan Africa, where practices such as delayed initiation of breastfeeding, premature cessation of exclusive breastfeeding, low dietary diversity, and inadequate meal frequency remain widespread [11]. Situating the economic burden of malnutrition within this behavioural and contextual landscape underscores the urgency of understanding current feeding practices and their determinants in order to design targeted, cost-effective interventions in the region.

While nutrition plays a pivotal role in promoting healthy child growth and preventing issues like stunting, underweight, and wasting, it’s crucial to recognize other risk factors. These factors encompass low birth weight, recurrent childhood infections, food insecurity, a higher number of children under five in a household, limited access to quality healthcare and education, and contextual elements, notably linked to inadequate water, sanitation, and hygiene (WASH) systems [12–14]. Poverty and food insecurity may limit access to diverse diets, affecting MDD and MAD. Similarly, maternal education influences feeding knowledge, while WASH affects food safety [11].

In the dynamic context of sub-Saharan Africa, where diverse socioeconomic, cultural, and environmental factors intersect, the significance of infant and child feeding practices cannot be overstated. Thus, in sub-Saharan Africa, infant and young child feeding practices are strongly shaped by intersecting socioeconomic, cultural, and environmental factors [15]. Caregivers often face food insecurity, gendered control of resources, and seasonal shortages that constrain access to diverse diets, while cultural norms and food taboos may restrict nutrient-rich foods for mothers and young children [16] Limited, inconsistent nutrition counselling within overstretched health systems further reduces caregivers’ capacity to adopt recommended practices, which partly accounts for the persistent suboptimal feeding behaviours despite well-documented benefits of appropriate feeding [17, 18] .

In 2002, the World Health Organization and UNICEF adopted the Global Strategy for Infant and Young Child Feeding (19). This strategy aimed to highlight the profound impact of feeding practices on the nutritional status, growth, development, health, and survival of infants and young children. Although the benefits of appropriate infant and young child feeding practices for linear growth are well documented [6, 20], suboptimal practices remain widespread in many sub-Saharan African settings [21, 22]. These behaviours are sustained by interacting factors, including cultural norms and food taboos, poverty and food insecurity that constrain access to diverse diets, limited maternal nutrition knowledge, and structural barriers such as inadequate WASH infrastructure. Understanding how these determinants shape everyday feeding practices is essential for situating child growth outcomes within the lived realities of sub-Saharan African households and for demonstrating the need and originality of the present multi-country analysis. Prior studies in SSA [13–16] identified low rates of MDD and MAD but lacked multi-country analysis of their association with growth outcomes.

Our study fills this gap by investigating children’s feeding practices [17] and how they affect growth outcomes in sub-Saharan Africa. Consequently, this study seeks to provide a nuanced understanding of children’s feeding practices and their association with growth outcomes in sub-Saharan Africa. Thus, we examined WHO-recommended practices: exclusive breastfeeding, age-appropriate feeding, dietary diversity (MDD), meal frequency (MMF), and acceptable diet (MAD). A child is considered to receive age-appropriate feeding he/she is not wasted, stunted or underweight. By unraveling the complex web of factors contributing to these outcomes, we aim to inform evidence-based interventions and policy recommendations tailored to the specific needs and circumstances of sub-Saharan African children. Findings could inform nutrition-sensitive policies: feeding guidelines, maternal health programs, and WASH infrastructure.

## Methods

### Datasets

In this study, we consider data from 174,281 children under the age of 5 from 30 sub-Saharan Africa countries. We identified the most recent Demographic and Health Surveys conducted from 2010 to 2022 and downloaded from the DHS website (http://dhsprogram.com) after being granted permission. We did not include datasets beyond 2022 but focused on 2010 to 2022 due to the following: (1) Only 3/30 SSA countries had released 2023–24 data during analysis (2022); (2) Surveys post-2022 used revised anthropometric tools, risking comparability and finally (3) Our study period (2010–2022) ensured consistency with pre-COVID trends. The Child’s Recode (KR File) dataset from each country was imported into Stata 18.0 [18] and pooled for further analysis. Further details on the DHS survey implementation and study design are described elsewhere [19].

## Study variables and child anthropometry measurements

Table 1 presents the description of the variables included in this study. The main outcome variables are (i) Wasting (weight-for-height, WHZ<-2), (ii) stunting (height-for-age, HAZ <-2), (iii) underweight (weight-for-age, WAZ <-2), and (iv) co-existence of wasting, stunting, and underweight. Across all countries, standardized DHS tools were used to measure anthropometric measurements, in line with the recommendations by the World Health Organisation (WHO) [20].

**Table 1:** Description of exploratory variables considered in the study

Variable	Variable description/classification
Low birth weight (LBW)	LBW defined as reported birthweight < 2500 g: Yes, no
Child gender	Sex: Male, female
Child age	Child age in months:<6, 6–11, 12–23,24–35,36–47,48–59
Exclusive breastfeeding	Exclusive breastfeeding for the first 6 months of life
Age-appropriate feeding	Age-appropriate feeding: Yes, no
MDD	Minimum dietary diversity: Yes, no
MMF	Minimum meal frequency: Yes, no
MAD	Minimum acceptable diet: Yes, no
Place of residence	Child place of residence: Urban, rural
Education	Mother education level: No education, primary, secondary and higher
Wealth	Household wealth quintile: Poorest, poorer, middle, richer, richest
Birth-order(ref:1)	Birth-order: 1, 2–3,4–5,6+
Body Mass Index (BMI)	Mother BMI: Underweight, Normal, overweight
Stature	Mother stature: Tall(> 145 cm), short(< 145 cm)
Marital Status (ref: Single)	Mother marital status: Single, married/union, divorced/widowed

## Analysis

DHS survey data-sets are often complex for two main reasons: (i) the use of stratified multistage cluster sampling, and (ii) unequal probabilities of selection from target populations for sampled elements, often as a result of oversampling of key subgroups. Hence, to account for the survey design and unequal probabilities of selection from target populations for sampled children, sampling weights were used to estimate proportions and their respective confidence intervals [21]. The descriptive analysis involved computation of child and maternal factors, presented in frequencies and percentages (see Table 2). We further analysed the weighted percentages of stunting, wasting, underweight, and their co-existence across included countries (see Table 3) and across children’s socio-demographic characteristics (see Table 4).

Children living in the same cluster may have similar feeding practices and more alike than children living in another cluster. Hence, our modeling approach should take into account the correlated nature of the data by introducing sampling clusters as random effects to account for a variety of situations, including cluster heterogeneity, and unobserved covariates within that cluster. As a result, to investigate the association between child feeding practices and other demographic factors with child weight and growth outcome, we employed a multilevel logistic regression model by considering survey clusters as a random effect, adjusting for different covariates presented. Below is the underlying analytical model:

1 $$\:\begin{array}{cccc}&\:\mathrm{l}\mathrm{o}\mathrm{g}\left(\frac{{p}_{ij}}{1-{p}_{ij}}\right)=\alpha\:F{P}_{ij}+{x}_{ij}^{{\prime\:}}\beta\:+{b}_{j}\end{array}$$

where $\:F{P}_{ij}\:$ represents feeding practices and $\:\boldsymbol{\alpha\:}\:$ is the parameter of interest. The vector $\:\boldsymbol{\beta\:}\:$ captures the effects of other covariates. The authors should then discuss the identification strategy for estimating $\:\boldsymbol{\alpha\:}$, including potential endogeneity concerns, omitted variable bias, or reverse causality.

To quantify the relative importance of survey clusters as a source of variation about child growth outcomes, cluster-level random effects variance was expressed in terms of Variance Partition Coefficients (VPC). The proportion of total variation of child growth outcome attributable to cluster-level random effect is computed as follows [22].

2 $$\:VP{C}_{{\sigma\:}_{b}^{2}}=\frac{{\sigma\:}_{b}^{2}}{{\frac{{\pi\:}^{2}}{3}+\sigma\:}_{b}^{2}}$$

where $\:{\sigma\:}_{b}^{2}$ is cluster-level random effect variance, and $\:\frac{{\pi\:}^{2}}{3}=3.29\:\:$ is individual level variance is equal to the variance of a logistic distribution [22]. We could not utilise multiple imputation to retain the data because the variable exclusive breastfeeding had > 80% missing data across the pooled dataset due to its age-specific nature (only applicable to children < 6 months). Multiple imputation assumes data are missing at random (MAR), which cannot be verified here. As a result of, high proportion of structurally missing data and the risk of generating implausible estimates, multiple imputation was deemed inappropriate. Therefore, the variable was excluded from the final analysis to minimise bias and preserve the validity of the findings. Meanwhile, missing data for covariates other than exclusive breastfeeding were minimal (< 5%) and handled via complete-case analysis. Covariates were selected via bivariable analysis (*p* < 0.2). Final models used *p* < 0.05 as the significance threshold. Given that multilevel logistic models lack a universal GOF test (e.g., the Hosmer-Lemeshow test is unsuitable for clustered data), we calculated Variance Partition Coefficients (VPCs) to quantify cluster-level variance (Table 5). We also assessed model stability via sensitivity analysis (excluding outliers). Both descriptive and inferential analyses were conducted using Stata 18.0 [18]. In this study, the statistical significance level is 0.05.

## Ethical consideration

As the study was based on secondary data, no further ethical approval was required from the authors beyond that provided by the Measure DHS Program. Details of the ethical standards reported by the Measure DHS Program are available at http://goo.gl/ny8T6X.

## Results

### Socio-demographic characteristics

In our study sample, the number of male and female children is nearly equal. Approximately 15% of them had low birth weight, and children who had a minimum diet diversity and a minimum acceptable diet were below 10%. Nearly one in four (26.5%) children had age-appropriate feeding practices. Table 2 summarizes the background characteristics of children and their mothers.

**Table 2:** Background characteristics of respondents (*n* = 174,281)

Background characteristics	Child-characteristics		Mother background characteristics		
	*n*	%		*n*	%
**Sex**			**Education**		
Male	87,815	50.4	No education	66,043	37.9
Female	86,466	49.6	Primary	58,302	33.5
**Age**			Secondary	42,195	24.2
< 6	18,993	10.9	Higher	7741	4.4
6–11	19,275	11.1	**Age at birth**		
12–23	36,498	20.9	< 20	25,706	14.7
24–35	33,632	19.3	20–34	122,665	70.4
36–47	33,875	19.4	35–49	25,910	14.9
48–59	32,006	18.4	**HH wealth**		
**Low-birth weight**			Poorest	39,356	22.6
No	148,533	85.2	Poorer	37,291	21.4
Yes	25,748	14.8	Middle	34,973	20.1
**Exclusive breastfeeding**			Richer	33,810	19.4
No	11,030	58.4	Richest	28,850	16.6
Yes	7841	41.6	**Birth-order**		
**Age-appropriate feeding**			1	37,119	21.3
No	128,025	73.5	2–3	62,344	35.8
Yes	46,256	26.5	4–5	39,815	22.8
**MDD-1**			6+	35,003	20.1
No	142,862	91.9	**BMI**		
Yes	12,548	8.1	Underweight	13,641	9.8
**MMF-2**			Normal weight	93,622	67.1
No	134,022	86.2	Overweight	32,275	23.1
Yes	21,388	13.8	**Stature**		
**MAD-3**			height>145 cm	137,360	98.3
No	149,980	96.5	Short(height<145 cm)	2342	1.7
Yes	5430	3.5	**Marital status**		
**Place of residence**			Single	10,699	6.1
Urban	54,676	31.4	Married/union	152,422	87.5
Rural	119,605	68.6	Divorced	11,160	6.4

**HH=Household

### Weighted percentage (95%CI) of stunting, wasting, underweight and their co-occurrence by country

Table 3 presents the prevalence of wasting, stunting, underweight and their co-existence by country. The results show that in all countries, the prevalence of stunting was higher as compared with the prevalence of wasting and underweight. Among our study samples from 30 countries, 6.94% (95%CI:6.79,7.09) were wasted, 32.17(95%CI:31.90,32.45) were stunted, 16.72%(95%CI:16.50,16.94) were underweight children, and 2.32% (95%CI:2.24,2.40) were wasted, stunted and underweight. The highest prevalence of wasting, underweight and co-occurrence of wasting, stunting and underweight was observed in Niger (Table 3).

**Table 3:** Weighted percentage (95%CI) of stunting, wasting, underweight and their co-occurrence by country

Country	Survey Year	*n*	Wasting	Stunting	Underweight	Co-occurrence of wasting, stunting, and underweight
Angola	2015-16	6,268	5.00(4.34, 5.77)	36.84(35.21, 38.49)	18.30(17.03, 19.64)	2.04(1.60, 2.59)
Burkina Faso	2010	6,532	15.78(14.84,16.78)	34.48(33.24,3574)	25.65(24.51,26.83)	4.56(4.04,5.14)
Burundi	2016-17	6,039	5.05(4.48,5.68)	55.74(54.36,57.12)	29.16(27.93,30.43)	3.44(2.98,3.98)
Congo DR	2013-14	8,059	7.95(7.15,8.83)	42.33(40.85,43.84)	22.37(21.13,23.66)	2.34(1.94,2.81)
Cote D’Ivoire	2011-12	3,200	7.79(6.66,9.08)	29.78(27.78,31.85)	14.71(13.20,16.35)	1.92(1.42,2.58)
Cameroon	2018-19	4,445	4.39(3.70,5.19)	28.77(27.23,30.37)	10.80(9.75,11.95)	1.25(0.91,1.70)
Ethiopia	2016	8,768	10.10(9.21,11.07)	38.31(36.81,39.82)	23.34(22.06,24.67)	3.08(2.60,3.64)
Gabon	2019-21	5,311	3.48(2.71,4.47)	13.74(12.44,15.15)	5.22(4.43,6.14)	0.56(0.33,0.96)
Ghana	2016	2,720	4.74(3.76,5.95)	17.93(16.23,19.76)	10.84(9.43,12.43)	1.35(0.91,2.00)
Gambia	2019-20	3,805	5.29(4.43,6.30)	16.89(15.43,18.44)	11.29(10.09,12.62)	1.44(1.00,2.07)
Guinea	2018	3,371	8.87(7.87,9.99)	30.69(29.01,32.42)	15.28(14.01,16.64)	1.75(1.34,2.27)
Kenya	2022	17,283	4.90(4.53,5.30)	17.26(16.52,18.02)	9.99(9.44,10.56)	1.39(1.20,1.60)
Liberia	2019-20	2,440	3.72(2.82,4.89)	28.81(26.46,31.28)	10.03(8.55,11.73)	1.54(0.99,2.39)
Lesotho	2014	1,312	3.05(2.20,4.21)	32.43(29.51,35.49)	10.82(8.99,12.97)	0.88(0.46,1.66)
Mali	2018	8,223	8.85(8.20,9.55)	26.55(25.50,27.63)	18.16(17.26,19.10)	2.89(2.52,3.32)
Malawi	2015-16	5,110	2.71(2.24,3.28)	36.54(34.93,38.19)	10.97(9.99,12.03)	0.93(0.67,1.29)
Mozambique	2011	9,313	5.95(5.38,6.58)	42.81(41.59,44.04)	14.94(14.05,15.87)	1.72(1.42,2.09)
Nigeria	2018	11,314	6.82(6.27,7.42)	36.43(35.38,37.49)	21.20(20.32,22.11)	3.17(2.78,3.62)
Niger	2012	4,771	18.10(16.87,19.41)	43.25(41.64,44.88)	36.16(34.60,37.75)	7.53(6.71,8.44)
Namibia	2013	1,558	7.63(6.31,9.21)	22.03(19.82,24.41)	13.66(11.84,15.71)	1.85(1.28,2.67)
Rwanda	2019-20	3,806	1.12(0.81,1.54)	32.95(31.39,34.56)	7.48(6.64,8.42)	0.42(0.26,0.69)
Sierra Leone	2019	4,100	5.57(4.77,6.49)	28.75(27.20,30.35)	13.13(12.03,14.31)	1.79(1.38,2.31)
Senegal	2019	5,524	8.03(7.22,8.91)	17.51(16.32,18.76)	13.96(12.91,15.08)	2.20(1.82,2.64)
Chad	2014-15	9,826	13.23(12.45,14.06)	39.60(38.43,40.79)	28.79(27.73,29.88)	5.27(4.76,5.83)
Togo	2013-14	3,185	6.77(5.89,7.78)	26.68(25.03,28.39)	15.73(14.40,17.16)	2.43(1.92,3.09)
Tanzania	2015-16	8,938	4.74(4.23,5.32)	34.03(32.86,35.22)	13.36(12.53,14.24)	1.53(1.26,1.87)
Uganda	2016	4,390	3.66(3.09,4.33)	28.17(26.67,29.72)	9.88(8.95,10.90)	1.05(0.76,1.44)
South Africa	2016	1,075	2.48(1.50,4.07)	25.41(22.09,29.04)	5.24(3.69,7.37)	0.50(0.12,2.01)
Zambia	2018	8,681	4.23(3.74,4.78)	34.39(33.20,35.61)	11.60(10.83,12.41)	1.00(0.77,1.29)
Zimbabwe	2015	4,914	3.53(2.98,4.16)	25.92(24.54,27.34)	7.84(7.02,8.75)	0.72(0.49,1.08)
**Total**	**-**	**174**,**281**	**6.94(6.79**,**7.09)**	**32.17(31.90**,**32.45)**	**16.72(16.50**,**16.94)**	**2.32(2.24**,**2.40)**

### Prevalence of wasting, stunting, underweight, and their co-occurrence among children in sub-Saharan Africa by different child socio-demographic characteristics

As illustrated in Table 4, most of the children were stunted compared with being wasted or underweight, across all socio-demographic characteristics. Notably, relatively more males were stunted (34.3%), as well as children between 24 and 35 months (41.1%), and those with low birth weight (40.7%). Relatedly, a significant proportion of children who were exclusively breastfed were stunted (15%), just as their colleagues without age-appropriate feeding (33.9%), those without the minimum dietary diversity (34.8%), those without minimum meal frequency (35.3%), those without minimum acceptable diet (34.6%) and the rural residents (36.0%).

**Table 4:** Prevalence of wasting, stunting, underweight and their co-occurrence among children in sub-Saharan Africa by different child socio-demographic characteristics

Background characteristics	Wasting		Stunting		Underweight		Coexistence	
	*n*	%	*n*	%	*n*	%	*n*	%
**Sex**								
Male	7028	8.0	30,129	34.3	16,056	18.3	2627	3.0
Female	5578	6.4	25,899	29.9	13,937	16.1	1603	1.9
**Age (months)**								
< 6	1669	8.8	2809	14.9	1859	9.9	145	0.8
6–11	2084	10.9	3690	19.3	2997	15.7	493	2.6
12–23	3359	9.2	12,988	35.7	6928	19.0	1575	4.3
24–35	1999	5.9	13,981	41.1	6542	19.2	882	2.6
36–47	1666	5.0	12,552	37.3	6003	17.8	617	1.8
48–59	1829	5.7	10,008	31.1	5664	17.6	518	1.6
**Low-birth weight**								
No	9676	6.5	45,431	30.6	22,804	15.4	2997	2.0
Yes	2930	11.3	10,597	40.7	7189	27.6	1233	4.7
**Exclusive breastfeeding**								
No	1125	10.2	1638	14.8	1272	11.5	113	1.0
Yes	544	7.0	1171	15.0	587	7.5	32	0.4
**Age-appropriate feeding**								
No	8312	6.5	43,588	33.9	22,566	17.6	2777	2.2
Yes	4294	9.4	12,440	27.2	7427	16.3	1453	3.2
**Minimum dietary diversity**								
No	10,146	7.1	49,903	34.8	26,503	18.5	3811	2.7
Yes	791	6.6	3316	27.8	1631	13.7	274	2.3
**Minimum meal frequency**								
No	8960	6.7	47,451	35.3	24,690	18.4	3382	2.5
Yes	1977	9.5	5768	27.6	3444	16.5	703	3.4
**Minimum acceptable diet**								
No	10,554	7.0	51,937	34.6	27,463	18.3	3963	2.6
Yes	383	7.4	1282	24.8	671	13.0	122	2.4
**Place of residence**								
Urban	3066	5.8	12,362	23.3	6148	11.6	810	1.5
Rural	9540	7.9	43,666	36.0	23,845	19.7	3420	2.8

### The odds of being wasted, stunted, underweight, and their coexistence in children with 95% confidence intervals under different factors

Table 5 presents the results of the association between different factors with the nutritional status of children under five in sub-Saharan African countries. The results revealed that conditional on the random effect, children with low birth weight were more likely to be wasted [AOR = 1.65, 95% CI = 1.57–1.74], stunted [AOR = 1.49, 95% CI = 1.44–1.54] and underweight [AOR = 1.91, 95% CI = 1.84–1.98]. Among children with the same random effect, females were observed to have lower odds of all three indicators as manifested in their co-existence (AOR = 0.59, 95% CI = 0.55–0.63). Children 12–23 months had the highest odds of experiencing the co-existence of stunting, wasting and underweight (AOR = 5.86, 95% CI = 4.94–6.95) relative to those below 6 months. Meanwhile, those who had exclusive breastfeeding had lower odds relative to those who did not have exclusive breastfeeding (AOR = 0.40, 95% CI = 0.27–0.59), however, children with age-appropriate feeding had higher odds of experiencing the co-existence of the three indicators (AOR = 1.68, 95% CI = 1.54–1.84).

**Table 5:** The odds of being wasted, stunted and underweight, and their coexistence in children with 95% confidence intervals under different factors

Factor	Wasting	Stunting	Underweight	Co-existence
	AOR (95%CI)	AOR (95%CI)	AOR (95%CI)	AOR (95%CI)
**Low-birth weight (ref: No)**				
Yes	1.65(1.57,1.74)	1.49(1.44,1.54)	1.91(1.84,1.98)	2.16(2.00,2.33)
**Sex (ref: Male)**				
Female	0.76(0.72,0.79)	0.79(0.77,0.81)	0.83(0.80,0.85)	0.59(0.55,0.63)
**Age (ref: <6 months)**				
6–11	1.26(1.18,1.35)	1.37(1.30,1.45)	1.7(1.60,1.81)	3.42(2.84,4.12)
12–23	1.05(0.98,1.11)	3.21(3.07,3.36)	2.16(2.05,2.28)	5.86(4.94,6.95)
24–35	0.64(0.60,0.69)	4.04(3.86,4.23)	2.19(2.07,2.31)	3.44(2.88,4.10)
36–47	0.53(0.50,0.57)	3.45(3.29,3.61)	1.99(1.89,2.11)	2.41(2.01,2.89)
48–59	0.62(0.58,0.66)	2.61(2.49,2.74)	1.96(1.86,2.08)	2.12(1.76,2.55)
**Exclusive breastfeeding** (ref: No)				
Yes	0.66(0.59,0.73)	1.01(0.93,1.10)	0.62(0.59,0.69)	0.40(0.27,0.59)
**Age-appropriate feeding** (ref: No)				
Yes	1.75(1.65,1.85)	0.75(0.72,0.77)	1.01(0.97,1.06)	1.68(1.54,1.84)
**Minimum dietary diversity** (ref: No)				
Yes	0.82(0.73,0.92)	0.97(0.92,1.03)	0.82(0.76,0.89)	0.86(0.72,1.04)
**Minimum meal frequency** (ref: No)				
Yes	1.15(1.07,1.23)	0.83(0.79,0.87)	0.91(0.86,0.96)	1.08(0.96,1.21)
**Minimum acceptable diet** (ref: No)				
Yes	1.01(0.85,1.19)	1.06(0.96,1.18)	1.09(0.96,1.24)	0.99(0.75,1.30)
**Place of residence** (ref: urban)				
Rural	1.00(0.94,1.07)	1.29(1.25,1.34)	1.19(1.14,1.24)	1.1(0.99,1.23)
**Education (**ref: No education**)**				
Primary	0.51(0.48,0.54)	0.88(0.86,0.91)	0.6(0.58,0.62)	0.51(0.47,0.56)
Secondary	0.47(0.44,0.50)	0.6(0.58,0.62)	0.46(0.44,0.48)	0.39(0.34,0.44)
Higher	0.45(0.38,0.53)	0.28(0.25,0.31)	0.29(0.25,0.33)	0.21(0.14,0.32)
**HH wealth** (ref: poorest)				
Poorer	0.85(0.80,0.91)	1.01(0.98,1.05)	0.95(0.91,0.99)	0.90(0.82,0.99)
Middle	0.90(0.84,0.96)	0.94(0.91,0.98)	0.92(0.88,0.96)	0.86(0.77,0.95)
Richer	0.96(0.90,1.03)	0.84(0.81,0.87)	0.88(0.84,0.92)	0.91(0.81,1.01)
Richest	0.98(0.89,1.07)	0.67(0.64,0.70)	0.80(0.75,0.85)	0.86(0.74,1.00)
**Birth-order** (ref:1)				
2–3	0.99(0.93,1.06)	0.99(0.96,1.03)	1.05(1.00,1.10)	1.01(0.91,1.12)
4–5	1.06(0.98,1.13)	1.04(1.00,1.08)	1.13(1.08,1.19)	1.14(1.02,1.27)
6+	1.09(1.02,1.17)	1.07(1.03,1.11)	1.21(1.15,1.27)	1.14(1.02,1.28)
**BMI** (ref: underweight)				
Normal weight	0.5(0.48,0.53)	0.86(0.82,0.89)	0.54(0.51,0.56)	0.45(0.41,0.49)
Overweight	0.33(0.30,0.36)	0.54(0.52,0.57)	0.28(0.26,0.29)	0.25(0.22,0.29)
**Stature** (ref: >145 cm)				
Short (< 145)	1.08(0.91,1.27)	3.04(2.76,3.34)	2.23(2.02,2.47)	1.59(1.27,1.99)
**Marital Status** (ref: Single)				
Married/union	1.17(1.03,1.32)	1.02(0.96,1.08)	1.12(1.04,1.22)	1.25(1.01,1.55)
Divorced	1.09(0.94,1.26)	1.13(1.05,1.22)	1.08(0.98,1.19)	1.25(0.98,1.61)
**Random effect**				
**Var(cluster) -**	**0.04(0.02**,**0.05)**	**0.03(0.02**,**0.04)**	**0.02(0.02**,**0.03)**	**0.05(0.03**,**0.09)**
**VPC**	**1.2%**	**0.9%**	**0.6%**	**1.5%**

Regarding education, children whose mothers had higher education had the lowest likelihood of coexistence of stunting, wasting, and underweight (AOR = 0.21, 95% CI = 0.14–0.32). Children of women in the middle and richest wealth quintiles also had lower odds. Meanwhile, among children with the same random effect, higher odds of coexistence of the three factors were observed among those with a sixth or higher birth order (AOR = 1.14, 95% CI = 1.02–1.28) and among those whose mothers are short in stature (AOR = 1.59, CI = 1.27–1.99). However, lower odds of co-existence of stunting, wasting, and underweight were observed among overweight children (AOR = 0.25, 95% 0.22–0.29). The variance partition coefficient (VPC) for wasting, stunting, underweight, and their coexistence attributed to differences between survey clusters is 1.2%, 0.9%,0.6% and 1.5%, respectively.

## Discussion

The findings from this multi-country study in sub-Saharan Africa provide crucial insights into the relationship between infant/child feeding practices and growth outcomes. These results shed light on the multifaceted nature of child malnutrition in the sub-regions and emphasize the importance of addressing specific factors to improve child health and nutrition. The high prevalence of stunting, which affects over 32% of children in the study sample across 30 countries, is a cause for concern. Relatedly, Kaldenbach et al. [23] have accentuated the double burden of stunting and a rise in obesity among children in South Africa. Stunting indicates chronic malnutrition and can have long-term adverse consequences on physical and cognitive development in children [24] It is essential to address the underlying causes of stunting, such as inadequate nutrition and poor maternal health outcomes, to break the cycle of malnutrition and promote optimal growth and development [25] .

The study revealed that children born with low birth weight (LBW) face significantly higher odds of experiencing wasting, stunting, underweight, and the coexistence of these conditions. These findings align with previous research emphasizing the critical role of LBW as a risk factor for child malnutrition [6–28]. The study’s consistency across different studies reinforces the validity and importance of this association. Similarly, other studies conducted in Malawi, Bangladesh, and India have also shown that socio-demographics, comorbidities, and nutritional characteristics are significantly associated with low birth weight [29–31]. Maternal comorbidities directly contribute to LBW and indirectly affect child malnutrition through maternal health and nutrition [32]. Variations in dietary habits and access to nutritional resources among pregnant women can lead to different LBW rates in various study settings. Interventions to improve maternal nutrition during pregnancy and adequate prenatal care can help address this issue and reduce the burden of child malnutrition [33].

Furthermore, gender disparity in child nutritional outcomes was significant, with female children having lower odds of wasting, stunting, underweight, and coexisting malnutrition than males. These findings are consistent with a study conducted in Pakistan that observed gender inequality in food intake; calories were a major cause of malnutrition among female children [34]. However, there are conflicting findings. For instance [35], reported that in Ethiopia, the chances of boys having stunted growth are higher than girls, while in the USA, it was reported that household food insecurity experienced by boys has lower odds of obesity than girls [36]. Possible reasons for variation in findings include differences in the definition and measurement of child malnutrition, sample characteristics, and contextual factors across studies. These findings highlight the need for targeted interventions considering gender-specific factors influencing child nutrition.

Age was a significant factor in children’s nutritional outcomes, with children aged 6–11 months more likely to experience wasting, stunting, underweight, and coexistence than children under 6 months. Similarly, children aged 12–23 months were more likely to experience stunting and underweight, while those aged 24–35 months were more likely to experience stunting and wasting. This indicates that different age groups are more prone to specific forms of undernutrition. These findings are consistent with [37] that low birthweight was a risk factor for wasting and stunting at 6 and 24 months, which are predicted by prior episodes of stunting and concurrent wasting-stunting. These findings across age groups imply that children’s nutritional needs differ at different developmental stages. Hence, there’s a need for early interventions to prevent and address child undernutrition.

Adequate feeding practices are paramount for promoting child nutrition. Our study found that exclusive breastfeeding reduced the odds of adverse outcomes, which is consistent with the findings of the WHO [38]. Despite growing evidence in support of exclusive breastfeeding (EBF) among infants in the first 6 months of birth, the debate over the optimal duration of EBF continues. Scholars have debated optimal durations for infants’ exclusive breastfeeding (EBF). The World Health Organization (WHO) recommends exclusive breastfeeding for the first 6 months of life as the optimal duration [38]. Some scholars have also argued that exclusive breastfeeding can provide additional health benefits for infants and contribute to optimal growth and development [39, 40]. However, Fufa & Laloto [41] reported a higher rate of hospital admission among infants who commence complementary feeding at 4 months. This implies that breastfeeding practices depend on several factors, including maternal and fetal factors as well as cultural and societal factors. Therefore, interventions emphasizing practical education may be required to facilitate exclusive breastfeeding and promote appropriate feeding practices during the critical early years of life.

Several factors are associated with children’s nutritional outcomes. The findings included higher maternal education levels, greater household wealth, and taller maternal stature were generally linked to improved child nutritional outcomes. These align with previous studies that reported a similar association. Specifically, studies have shown that mothers who had no formal education, food insecure, and children who had no feeding plate were associated with stunting, undernutrition [42]. Additionally, children living in rural areas were more likely to experience stunting and underweight as also reported by some prior studies [43, 44]. Some studies also indicate that determinants of malnutrition include biological, social, cultural, economic, and morbidity factors, including child-related (child age, birth weight, and birth order), parental/household-related (maternal education, nutritional status, family size, household income), and community or area-related factors [45-48–]. While feeding practices (e.g., MAD) were significant, low birth weight and maternal short stature were dominant predictors, necessitating integrated interventions. These findings highlight the importance of addressing the social determinants of child malnutrition, including maternal education and household economic status. Therefore, comprehensive interventions need to be tailored to specific contexts and populations to be effective.

### Strengths and limitations of the study

The study has several outstanding strengths worth mentioning. First, it presents robust empirical findings from 30 countries. Underpinned by sound analytical procedure, the study is representative of the children included. Also, it adds to the scanty literature on the association between children’s feeding and their well-being. Despite the study’s strengths, it is cross-sectional and does not establish causality between feeding practices and growth outcomes in sub-Saharan Africa. Besides, the missing datasets were excluded. We could not utilise multiple imputation to retain the data because the variable exclusive breastfeeding had > 80% missing data across the pooled dataset due to its age-specific nature (only applicable to children < 6 months). Multiple imputation assumes that data are missing at random (MAR), which cannot be verified here. As a result, the exclusion minimized bias from implausible imputations.

## Conclusion

This comprehensive study involving 30 countries in sub-Saharan Africa provides critical insights into the nutritional status of children, highlighting persistently high rates of malnutrition — particularly stunting — and its long-term consequences. *Our analysis* **confirms that suboptimal feeding practices (e.g.**,** non-exclusive breastfeeding and inappropriate age-appropriate feeding) significantly predict adverse growth outcomes**. *Low birth weight and maternal short stature were critical covariates. While missing data limited* the analysis of exclusive breastfeeding, our findings on MDD/MMF/MAD underscore the need to prioritize **context-specific feeding interventions** *alongside maternal health. Future studies should address data gaps in infant feeding metrics.* The findings underscore significant disparities between countries and reveal links between low birth weight and later growth challenges, reinforcing the need for targeted interventions. In particular, strategies that improve complementary feeding practices, dietary diversity, and meal frequency can directly address these disparities and mitigate the nutritional disadvantages that begin at birth. These findings should guide policymakers, healthcare professionals, and stakeholders in developing targeted strategies to improve complementary feeding practices, support healthy growth, and enhance the overall well-being of children in the region. Through conscious, concerted efforts, the burden of malnutrition can be alleviated to ensure a brighter future for children in sub-Saharan Africa. Context-specific interventions prioritizing appropriate infant and child feeding practices, alongside maternal nutrition and prenatal care, are crucial in mitigating the impact of malnutrition on children in the region. This study is not devoid of limitations. Reliance on cross-sectional DHS data collected in different years across countries (for example, 2012 for Niger versus 2018–2020 for many others) constrains any causal interpretation of the observed associations between feeding practices and growth outcomes, and cross-country differences may partly reflect temporal variation in contextual conditions rather than stable structural disparities. Future longitudinal or quasi-experimental studies incorporating more granular information on feeding behaviours are required to elucidate these pathways more robustly and to strengthen the evidentiary basis for policy and programme design.

## Data Availability

The survey datasets are freely available to download via https://dhsprogram.com/data/dataset_admin/index.cfm.

## Declarations

domain.

## References

[1] Food and Agriculture Organization (FAO). F and A. Building climate resilience for food security and nutrition. The state of food security and nutrition in the world. FAO Roma; 2018.

[2] Zoleko-Manego R, Mischlinger J, Dejon-Agobé JC, Basra A, Mackanga JR, Akerey Diop D, et al. Birth weight, growth, nutritional status and mortality of infants from Lambaréné and Fougamou in Gabon in their first year of life. PLoS ONE. 2021;16(2):e0246694. 33561169 10.1371/journal.pone.0246694 PMC7872243

[3] Saleem J, Zakar R, Bukhari GMJ, Fatima A, Fischer F. Developmental delay and its predictors among children under five years of age with uncomplicated severe acute malnutrition: a cross-sectional study in rural Pakistan. BMC Public Health. 2021;21(1):1–10. 34266406 10.1186/s12889-021-11445-w PMC8281691

[4] Getaneh T, Negesse A, Dessie G, Desta M, Assemie MA, Tigabu A. Predictors of malnutrition among pregnant women in ethiopia: a systematic review and meta-analysis. Hum Nutr Metab. 2021;26:200131. 10.1155/2021/6551526 PMC8654570 34901276

[5] Dewey KG, Begum K. Long-term consequences of stunting in early life. Matern Child Nutr. 2011;7:5–18. 21929633 10.1111/j.1740-8709.2011.00349.x PMC6860846

[6] Zegeye AF, Tamir TT, Asmamaw DB, Bitew DA, Fentie EA, Terefe B et al. Minimum dietary diversity and its determinants among lactating mothers in five Sub-Saharan African countries: A multilevel analysis. Plos One [Internet]. 2025 [cited 2026 Jan 7];20(3):e0308590. Available from: https://journals.plos.org/plosone/article?id=10.1371/journal.pone.0308590 10.1371/journal.pone.0308590 PMC11882081 40043062

[7] World Health Organization. Malnutrition. [Internet]. 2023 [cited 2023 Sept 24]. Available from: https://www.who.int/health-topics/malnutrition#tab=tab_1

[8] Büttner N, Heemann M, De Neve JW, Verguet S, Vollmer S, Harttgen K. Economic Growth and Childhood Malnutrition in Low- and Middle-Income Countries. JAMA Netw Open [Internet]. 2023 Nov 9 [cited 2026 Jan 7];6(11):e2342654. Available from: https://jamanetwork.com/journals/jamanetworkopen/fullarticle/2811562 10.1001/jamanetworkopen.2023.42654 PMC10636637 37943556

[9] Fernandes C, Le MT. Physical And Cognitive Development Delays As A Result Of Malnutrition Early In Life, Having Individual, Societal, And Economic Impacts. Child Refug Migr Health Man Health Prof [Internet]. 2021 [cited 2026 Jan 7];99. Available from:

[10] Mandela N. The slow violence of malnutrition. South Afr Child Gauge 2020 [Internet]. 2020 [cited 2026 Jan 7];24. Available from: https://www.researchgate.net/profile/Safura-Abdool-Karim/publication/356377734_Double_burden_and_double_duty_Government_action_required_to_improve_child_nutrition/links/61974bc03068c54fa5000359/Double-burden-and-double-duty-Government-action-required-to-improve-child-nutrition.pdf#page=25

[11] Wuehler SE, Hess SY, Brown KH. Accelerating improvements in nutritional and health status of young children in the Sahel region of Sub-Saharan Africa: review of international guidelines on infant and young child feeding and nutrition. Matern Child Nutr [Internet]. 2011 Apr [cited 2026 Jan 7];7(s1):6–34. Available from: https://onlinelibrary.wiley.com/doi/10.1111/j.1740-8709.2010.00306.x 10.1111/j.1740-8709.2010.00306.x PMC6860809 21410888

[12] De Onis M, Branca F. Childhood stunting: a global perspective. Matern Child Nutr. 2016;12:12–26. 27187907 10.1111/mcn.12231 PMC5084763

[13] Chowdhury R, Sinha B, Sankar MJ, Taneja S, Bhandari N, Rollins N, et al. Breastfeeding and maternal health outcomes: a systematic review and meta-analysis. Acta Paediatr. 2015;104:96–113. 26172878 10.1111/apa.13102 PMC4670483

[14] Stewart CP, Iannotti L, Dewey KG, Michaelsen KF, Onyango AW. Contextualising complementary feeding in a broader framework for stunting prevention. Matern Child Nutr. 2013;9:27–45. 24074316 10.1111/mcn.12088 PMC6860787

[15] Chakona G. Social circumstances and cultural beliefs influence maternal nutrition, breastfeeding and child feeding practices in South Africa. Nutr J [Internet]. 2020 Dec [cited 2026 Jan 7];19(1):47. Available from: https://nutritionj.biomedcentral.com/articles/10.1186/s12937-020-00566-4 10.1186/s12937-020-00566-4 PMC7240933 32434557

[16] Tugume P, Mustafa AS, Walusansa A, Ojelel S, Nyachwo EB, Muhumuza E et al. Unravelling taboos and cultural beliefs associated with hidden hunger among pregnant and breast-feeding women in Buyende district Eastern Uganda. J Ethnobiol Ethnomedicine [Internet]. 2024 May 2 [cited 2026 Jan 7];20(1):46. Available from: https://ethnobiomed.biomedcentral.com/articles/10.1186/s13002-024-00682-z 10.1186/s13002-024-00682-z PMC11064283 38693532

[17] Sanni SM. Nutrition Transition in West Africa: Public Health Challenges, Socioeconomic Impacts, And Regional Policy Interventions. Cent Med [Internet]. 2025 [cited 2026 Jan 7];1(1). Available from: https://centerofsciencepress.com/COM/article/view/9

[18] Berti C, Socha P, Infant. and young child feeding practices and health [Internet]. Vol. 15, Nutrients. MDPI; 2023 [cited 2026 Jan 7]. p. 1184. Available from: https://www.mdpi.com/2072-6643/15/5/1184 10.3390/nu15051184 PMC10005283 36904182

[19] Organization WH, UNICEF. Global strategy for infant and young child feeding. World Health Organization; 2003.

[20] Yesuf NN, Mekonnen EG, Takele WW. Minimum dietary diversity and associated factors among young infants and children living in the most productive area of Amhara region, Addis Zemen town: a community-based cross-sectional study. Int J Afr Nurs Sci [Internet]. 2021 [cited 2026 Jan 7];14:100279. Available from: https://www.sciencedirect.com/science/article/pii/S2214139121000020

[21] Ogbo FA, Agho K, Ogeleka P, Woolfenden S, Page A, Eastwood J et al. Infant feeding practices and diarrhoea in sub-Saharan African countries with high diarrhoea mortality. PloS One [Internet]. 2017 [cited 2026 Jan 7];12(2):e0171792. Available from: https://journals.plos.org/plosone/article?id=10.1371/journal.pone.0171792 10.1371/journal.pone.0171792 PMC5305225 28192518

[22] Tekeba B, Tamir TT, Workneh BS, Wassie M, Terefe B, Ali MS et al. Prevalence and determinants of unhealthy feeding practices among young children aged 6–23 months in five sub-Saharan African countries. PloS One [Internet]. 2025 [cited 2026 Jan 7];20(1):e0317494. Available from: https://journals.plos.org/plosone/article?id=10.1371/journal.pone.0317494 10.1371/journal.pone.0317494 PMC11734912 39813280

[23] Christian AK, Afful-Dadzie E, Marquis GS. Infant and young child feeding practices are associated with childhood anaemia and stunting in sub-Saharan Africa. BMC Nutr. 2023;9(1):9. 36627696 10.1186/s40795-022-00667-9 PMC9832766

[24] StataCorp L. Stata statistical software: Release 15 (2017). Coll Stn TX StataCorp LP. 2017.

[25] Corsi DJ, Neuman M, Finlay JE, Subramanian SV. Demographic and health surveys: a profile. Int J Epidemiol. 2012;41(6):1602–13. 23148108 10.1093/ije/dys184

[26] Organization WH. WHO child growth standards: length/height-for-age, weight-for-age, weight-for-length, weight-for-height and body mass index-for-age: methods and development. World Health Organization; 2006.

[27] Carle AC. Fitting multilevel models in complex survey data with design weights: recommendations. BMC Med Res Methodol. 2009;9:1–13. 19602263 10.1186/1471-2288-9-49 PMC2717116

[28] Leyland AH, Groenewegen PP. Multilevel modelling for public health and health services research: health in context. Springer Nature; 2020. 33347097

[29] Kaldenbach S, Engebretsen IM, Haskins L, Conolly C, Horwood C. Infant feeding, growth monitoring and the double burden of malnutrition among children aged 6 months and their mothers in KwaZulu-Natal, South Africa. Matern Child Nutr. 2022;18(1):e13288. 34845831 10.1111/mcn.13288 PMC8710097

[30] Ekholuenetale M, Barrow A, Ekholuenetale CE, Tudeme G. Impact of stunting on early childhood cognitive development in benin: evidence from demographic and health survey. Egypt Pediatr Assoc Gaz. 2020;68(1):1–11.

[31] Quamme SH, Iversen PO. Prevalence of child stunting in Sub-Saharan Africa and its risk factors. Clin Nutr Open Sci. 2022;42:49–61.

[32] Prendergast AJ, Humphrey JH. The stunting syndrome in developing countries. Paediatr Int Child Health. 2014;34(4):250–65. 25310000 10.1179/2046905514Y.0000000158 PMC4232245

[33] Rahman MS, Howlader T, Masud MS, Rahman ML. Association of low-birth weight with malnutrition in children under five years in bangladesh: do mother’s education, socio-economic status, and birth interval matter? PLoS ONE. 2016;11(6):e0157814. 27355682 10.1371/journal.pone.0157814 PMC4927179

[34] Aboagye RG, Ahinkorah BO, Seidu AA, Frimpong JB, Archer AG, Adu C, et al. Birth weight and nutritional status of children under five in sub-Saharan Africa. PLoS ONE. 2022;17(6):e0269279. 35679306 10.1371/journal.pone.0269279 PMC9182265

[35] Jana A, Dey D, Ghosh R. Contribution of low birth weight to childhood undernutrition in india: evidence from the National family health survey 2019–2021. BMC Public Health. 2023;23(1):1–14. 37438769 10.1186/s12889-023-16160-2 PMC10337105

[36] Ntenda PAM. Association of low birth weight with undernutrition in preschool-aged children in Malawi. Nutr J. 2019;18(1):1–15. 31477113 10.1186/s12937-019-0477-8 PMC6719380

[37] Mamidi RS, Banjara SK, Manchala S, Babu CK, Geddam JB, Boiroju NK, et al. Maternal Nutrition, body composition and gestational weight gain on low birth weight and small for gestational Age—A cohort study in an Indian urban slum. Children. 2022;9(10):1460. 36291396 10.3390/children9101460 PMC9600910

[38] UNICEF. 2021. Maternal nutrition. [Internet]. 2021. Available from: https://www.unicef.org/nutrition/maternal

[39] Shafiq A, Hussain A, Asif M, Jameel A, Sadiq S, Kanwel S. Determinants of gender disparity in nutritional intake among children in pakistan: evidence from PDHS. Children. 2021;9(1):7. 35053635 10.3390/children9010007 PMC8774664

[40] Samuel A, Osendarp SJ, Feskens EJ, Lelisa A, Adish A, Kebede A, et al. Gender differences in nutritional status and determinants among infants (6–11 m): a cross-sectional study in two regions in Ethiopia. BMC Public Health. 2022;22(1):401. 35219315 10.1186/s12889-022-12772-2 PMC8881837

[41] Bae JH, Choi JH. Gender disparities in childhood obesity and household food insecurity. Nutrition. 2021;87:111190. 33773405 10.1016/j.nut.2021.111190

[42] Kohlmann K, Sudfeld CR, Garba S, Guindo O, Grais RF, Isanaka S. Exploring the relationships between wasting and stunting among a cohort of children under two years of age in Niger. BMC Public Health. 2021;21(1):1–9. 34548050 10.1186/s12889-021-11689-6 PMC8454021

[43] Amare ZY, Ahmed ME, Mehari AB. Determinants of nutritional status among children under age 5 in ethiopia: further analysis of the 2016 Ethiopia demographic and health survey. Glob Health. 2019;15:1–11. 10.1186/s12992-019-0505-7 PMC6836473 31694661

[44] Khan MN, Islam MM. Effect of exclusive breastfeeding on selected adverse health and nutritional outcomes: a nationally representative study. BMC Public Health. 2017;17:1–7. 29162064 10.1186/s12889-017-4913-4 PMC5697409

[45] World Health Organization. Exclusive breastfeeding for six months best for babies everywhere. [Internet]. 2011 [cited 2023 Sept 24]. Available from: https://www.who.int/news/item/15-01-2011-exclusive-breastfeeding-for-six-months-best-for-babies-everywhere

[46] Hossain S, Mihrshahi S. Exclusive breastfeeding and childhood morbidity: A narrative review. Int J Environ Res Public Health. 2022;19(22) 10.3390/ijerph192214804 PMC9691199 36429518

[47] Gupta S, Agarwal R, Aggarwal KC, Chellani H, Duggal A, Arya S, et al. Complementary feeding at 4 versus 6 months of age for preterm infants born at less than 34 weeks of gestation: a randomised, open-label, multicentre trial. Lancet Glob Health. 2017;5(5):e501–11. 28395845 10.1016/S2214-109X(17)30074-8 PMC5388893

[48] Fufa DA, Laloto TD. Factors associated with undernutrition among children aged between 6–36 months in semien bench district. Ethiopia Heliyon. 2021;7(5). 10.1016/j.heliyon.2021.e07072 PMC8141868 34041409

[49] Akombi BJ, Agho KE, Merom D, Renzaho AM, Hall JJ. Child malnutrition in sub-Saharan africa: A meta-analysis of demographic and health surveys (2006–2016). PLoS ONE. 2017;12(5):e0177338. 28494007 10.1371/journal.pone.0177338 PMC5426674

[50] Vilar-Compte M, Burrola-Méndez S, Lozano-Marrufo A, Ferré-Eguiluz I, Flores D, Gaitán-Rossi P, et al. Urban poverty and nutrition challenges associated with accessibility to a healthy diet: a global systematic literature review. Int J Equity Health. 2021;20:1–19. 33472636 10.1186/s12939-020-01330-0 PMC7816472

[51] Katoch OR. Determinants of malnutrition among children: A systematic review. Nutrition. 2022;96:111565. 35066367 10.1016/j.nut.2021.111565

[52] Obasohan PE, Walters SJ, Jacques R, Khatab K. Risk factors associated with malnutrition among children under-five years in sub-Saharan African countries: a scoping review. Int J Environ Res Public Health. 2020;17(23):8782. 33256022 10.3390/ijerph17238782 PMC7731119

[53] Tette E, Sifah EK, Nartey ET, Nuro-Ameyaw P, Tete-Donkor P, Biritwum RB. Maternal profiles and social determinants of malnutrition and the mdgs: what have we learnt? BMC Public Health. 2016;16(1):1–11. 26935849 10.1186/s12889-016-2853-z PMC4776384

